# Electrospun Nanofibers Hybrid Wrinkled Micropyramidal Architectures for Elastic Self-Powered Tactile and Motion Sensors

**DOI:** 10.3390/nano13071181

**Published:** 2023-03-26

**Authors:** Zhenpeng Cao, Xiuru Xu, Chubin He, Zhengchun Peng

**Affiliations:** Center for Stretchable Electronics and NanoSensors, Key Laboratory of Optoelectronic Devices and Systems of Ministry of Education and Guangdong Province, College of Physics and Optoelectronic Engineering, Shenzhen University, Shenzhen 518000, Chinazcpeng@szu.edu.cn (Z.P.)

**Keywords:** electrospun nanofibers, tactile sensors, hydrogels, stretchable

## Abstract

Conformable, sensitive, long-lasting, external power supplies-free multifunctional electronics are highly desired for personal healthcare monitoring and artificial intelligence. Herein, we report a series of stretchable, skin-like, self-powered tactile and motion sensors based on single-electrode mode triboelectric nanogenerators. The triboelectric sensors were composed of ultraelastic polyacrylamide (PAAm)/(polyvinyl pyrrolidone) PVP/(calcium chloride) CaCl_2_ conductive hydrogels and surface-modified silicon rubber thin films. The significant enhancement of electrospun polyvinylidene fluoride (PVDF) nanofiber-modified hierarchically wrinkled micropyramidal architectures for the friction layer was studied. The mechanism of the enhanced output performance of the electrospun PVDF nanofibers and the single-side/double-side wrinkled micropyramidal architectures-based sensors has been discussed in detail. The as-prepared devices exhibited excellent sensitivity of a maximum of 20.1 V/N (or 8.03 V/kPa) as tactile sensors to recognize a wide range of forces from 0.1 N to 30 N at low frequencies. In addition, multiple human motion monitoring was demonstrated, such as knee, finger, wrist, and neck movement and voice recognition. This work shows great potential for skin-like epidermal electronics in long-term medical monitoring and intelligent robot applications.

## 1. Introduction

Owing to the burgeoning market demand for soft electronics for the next generation of intelligence, stretchable skin-like devices have gained considerable attention [1]. Conformable wearable electronics enable the highly potential applications in human motion sensing [2,3], human–machine interface [4], medical detection [5], and soft robots [6]. Triboelectric nanogenerators (TENG)-based electronics have been considered as one of the solutions that can convert low-frequency and mechanical movement into electrical energy without external power supplies [7,8,9]. However, skin-like triboelectric sensors are facing the challenges of sensor versatility, elasticity, flexibility, conformity, sensitivity, durability, and so forth. Thus, the investigation of both high-performance stretchable conductive electrodes and dielectric friction layers for the triboelectric sensors is urgent. Recently, considerable efforts have been dedicated to improving their performances. On the one hand, stretchable electrode materials were obtained by mixing the elastomers (thermoplastic polyurethanes, silicone rubber) with typical conductive materials such as liquid metals [10,11], silver nanowires [12], graphene [13], and carbon nanotubes [14]. However, they normally exhibit poor extensibility and are quite rigid. Conductive hydrogels are good candidates for their softness, large elasticity, and excellent conductivity [15,16]. On the other hand, various high-dielectric materials such as nylon 6′6, polytetrafluoroethylene (PTFE), and PVDF have been studied to enhance the output sensing performance of triboelectric devices [13,17]. Among them, PVDF is an excellent candidate for its strong capability to gain electrons and excellent piezoelectricity for triboelectric devices. Generally, PVDF show at least four crystal phases, and the polar β phase showed the highest piezoelectric performance among the other crystal phases [18]. To obtain the high-performance β phase of PVDF, compared with the high-temperature annealing method, the electrospinning technique is a low-cost, highly efficient method to polarize the PVDF nanofibers from the α phase to the β phase via ten thousand volts of the electric field force. For instance, Vanjari and co-workers have reported a high efficiency TENG device from PVDF and silk faction materials with a peak short-circuit current of 11.3 uA and open-circuit voltage of 611 V [19]. Guo and co-workers successfully prepared TENG devices from electrospun β phase PVDF fibers. The β phase content of the optimized PVDF fiber by electrospinning method reached 91.87 ± 0.61%. The PVDF single fiber device can exhibit a voltage of 6.1 mV and a power of 3.52 pW at 0.5 Hz [20]. Furthermore, the output performance of triboelectric devices can be improved by increasing the surface morphology, such as surface roughness and surface area, and by building various surface microstructures and so on. Wen and co-workers have systematically studied the influence of surface roughness on the performance of triboelectric nanogenerators [21]. Padhan and co-workers synthesized mechanochemical synthesis ferromagnetic NiO-Ti-based nanocomposites and used them as a positive triboelectric layer to enhance the performance of the TENG devices and obtained a voltage of 62 V and a current of 250 nA [22]. Hajra and co-workers reported a ZIF-8 (HG)/Kapton-based dual-mode TENG device with high output performance owing to its excellent surface potential and surface roughness. The device can generate a maximum voltage of 150 V and a current of 4.95 µA [23].

This work demonstrates a simple, low-cost route for fabricating an encapsulated single-electrode-mode triboelectric sensor, which involves an ultrastretchable and conductive hydrogel as the electrode and surface-modified high-efficiency elastic thin films as the fraction layer. Polyvinyl pyrrolidone (PVP) and calcium chloride (CaCl_2_) were used to adjust the hydrogel polymer network mechanically and electrically. In addition, electrospun polyvinylidene fluoride (PVDF) nanofibers hybrid wrinkled micropyramidal architectures for the friction layer were analyzed and discussed. The measured tactile response sensitivity of our friction-surface-modified sensors was up to 20.1 V/N (or 8.03 V/kPa), and the optimum output voltage was about 4.3 times that of the unmodified devices. The present work may lead to a new method for fabricating high-performance tactile and motion triboelectric sensors.

## 2. Materials and Methods

**Figure 1:**
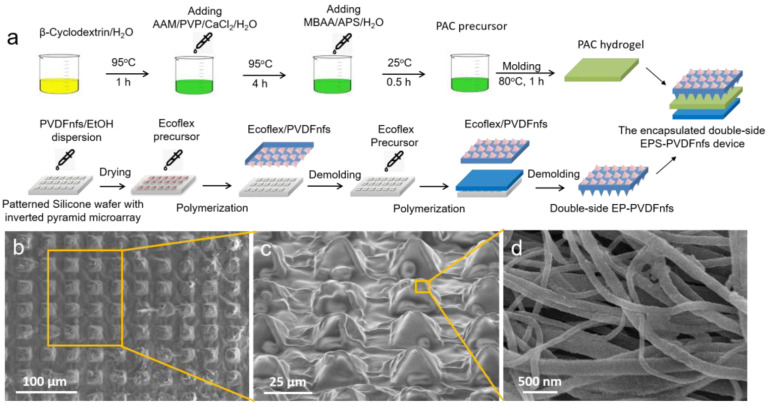
(**a**) Steps in the fabrication of the double-side EPS-PVDFnfs device. (**b**–**d**) Low- to high-resolution scanning electron microscopy images showing top-view and side-view with wrinkled micropyramid units and the surface PVDF nanofibers of the double-side EP-PVDFnfs thin film.

The schematic illustration shown in Figure 1a is the fabrication process strategy both of conductive hydrogels and the encapsulated triboelectric devices. All the chemicals were purchased from Aladdin Chem. Technology Co., Ltd. (Shanghai, China), and no further purification was applied before use.

### 2.1. Fabrication of PAAm/PVP/CaCl_2_ Conductive Hydrogels

We added 0.5 g cyclodextrin to a glass bottle with 5.0 g deionized (DI) water and stirred at 95 °C for 30min. Next, 1.0 g acrylamide (AAm) monomer, 0.02 g polyvinyl pyrrolidone (PVP, Mn = 1,300,000), and 0.02 g calcium chloride (CaCl_2_) were weighed and added to the mixer with continuous stirring at 95 °C for 4 h. Then the mixture was cooled down to room temperature. After that, we added 0.5 mg 4,4′-Diaminodiphenylmethane- 3,3′-dicarboxylic acid (MBAA) as the cross-linking agent and 0.03 g ammonium persulfate (APS) as the initiator to the mixer with continuous stirring for another 15 min at room temperature (RT). Finally, we centrifugal defoamed and templated it with thermally polymerizing at 80 °C for 1 h, then successfully obtained the as-prepared PAAm/PVP/CaCl_2_ (PAC) hydrogels and cut them into several pieces of the size 2.0 × 2.0 cm^2^.

### 2.2. Fabrication of the Single-Side/Double-Side Elastic Thin Films with Wrinkled Micropyramid Arrays

Firstly, 0.2 g of PVDF nanofibers film via electrospinning (with a voltage of 18 kV) was cut into small pieces, emerged in 10 mL of ethanol (EtOH), and then homogenized to form a 20 mg/mL dispersion with a homogenizer (IKA T18 basic, Wilmington, NC, USA). Then, the 20 mg/mL PVDF nanofibers/EtOH dispersion was dip-coated onto the 2.2 × 2.2 cm^2^ patterned silicon wafer with uniformly distributed inverted pyramid microstructures (20 μm long and wide, 10 μm high, with 20 μm gap) and transferred to a 100 °C heating plate for 10 min to evaporate the solvent completely. Secondly, we spin-coated silicone rubber precursor (Ecoflex 00-30, Smooth-On, Inc, Willow Lane East, TX, USA) onto the wafer at 500 rpm. We heated it at 80 °C for 30 min to obtain an approximately 200 μm-thick thin film. The Ecoflex thin film with electrospun PVDF nanofibers-covered pyramid microstructures array on a single side (named single-side EP-PVDFnfs thin film) were fabricated. Then we also fabricated the double-side EP-PVDFnfs thin film. We peeled off the as-prepared single-side EP-PVDFnfs thin film with microstructures array carefully. Then the same patterned silicon wafer was used to spin-coat another thin film with the pyramid microstructures array from Ecoflex at the same condition. The previous single-side EP-PVDFnfs thin film was put on the top of the as-prepared Ecoflex with the wafer and heated at 80 °C for 30 min for thermal polymerization. Finally, the Ecoflex thin films with electrospun PVDF nanofibers-covered pyramid microstructures array on both sides (double-side EP-PVDFnfs thin film) were peeled off and successfully fabricated.

### 2.3. Fabrication of the Self-Powered Piezoelectric Sensors

Firstly, we spin-coated a thin film from the Ecoflex precursor onto a silicon wafer at 500 rpm and heated it at 80 °C for 40 min to obtain an Ecoflex thin film with a smooth surface as the substrate. Then, we transferred a piece of 2.0 × 2.0 cm^2^ as-prepared PAC conductive hydrogel and a piece of double-side EP-PVDFnfs thin film on the top (keep the side with PVDF nanofibers of thin film on the top) accordingly. Copper tapes were used as the electrodes between the substrate and the gel. Next, we used a small amount of Ecoflex precursor to seal around the sample and thermally polymerized it at 80 °C for 30 min for the encapsulation. Finally, the self-powered triboelectric nanogenerators sensor with a structure of Ecoflex/PAC conductive hydrogel/double-side EP-PVDFnfs was successfully obtained and named the double-side EPS with PVDFnfs. We also prepared the sensors of Ecoflex/PAC conductive hydrogel/single-side EP-PVDFnfs (named single-side EPS with PVDFnfs) and Ecoflex/PAC conductive hydrogel/single-side EP (single-side EPS without PVDFnfs) with similar methods.

### 2.4. Characterization and Methods

The as-prepared samples were analyzed by Fourier infrared spectroscopy (FTIR, PerkinElmer, Inc., Waltham, MA, USA). The surface morphology of the films was characterized by scanning electron microscopy (SEM, SHIMADZU SSX-550, Tokyo, Japan). A universal material test instrument (E1000, Instron, Boston, MA, USA) and a digital multimeter (34465A,102 Keysight Technologies, Santa-Rosa, CA, USA) were used to test the mechanical and electrical performance. The output voltage was measured by a digital oscilloscope (Model ZDS2024B Plus, ZLG corp., Guangzhou, China) with 100:1 and 10:1 oscilloscope probes. The short-circuit current was tested by a current preamplifier (SR570, Stanford Research Systems, Sunnyvale, CA, USA).

## 3. Results

**Figure 2:**
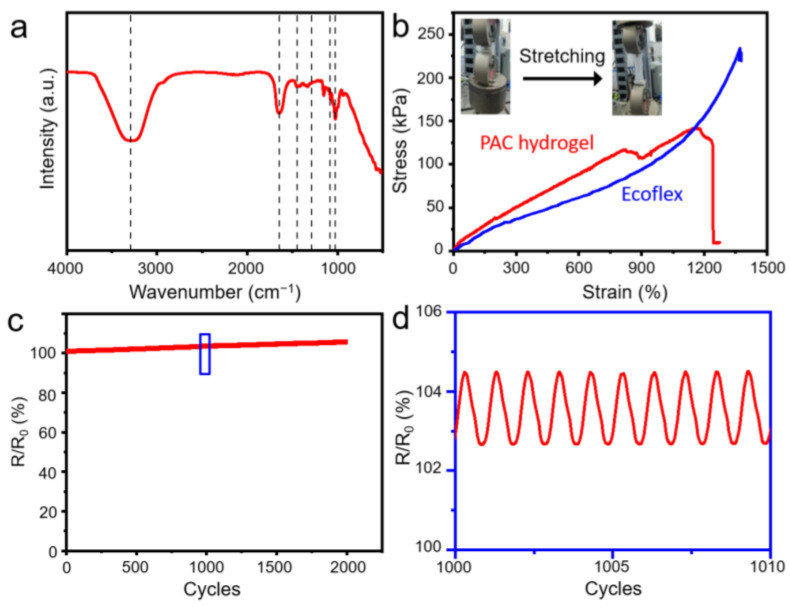
(**a**) FTIR spectra of the PAC hydrogels. (**b**) Strain–stress test of the PAC hydrogels and Ecoflex thin film. Durability loading–unloading test of PAC hydrogel for (**c**) over 2000 cycles at 2 Hz and (**d**) the high-resolution graph from the 1000th to the 1010th cycles.

The as-prepared self-powered piezoelectric sensors were based on a single-electrode triboelectric device. The structure of the sensor was schematically presented in Figure 1a. In this paper, we have electrospun the polymer PVDF into nanofibers and transferred the electrospun PVDF nanofibers onto the surface of the Ecoflex friction thin films with wrinkled micropyramidal architectures after dip-coating and polymerization. The surface morphology of the as-prepared single-side EP-PVDFnfs is characterized in Figure 1b–d. Wrinkled micropyramid arrays with hierarchical structures can be observed. The low-resolution microstructure in Figure 1b shows that the diameter of micropyramidal structures was 20 μm long and wide, 10 μm high, with a 25 μm period approximately, with a wrinkled and rough surface. From the high-resolution side-view SEM image, we can observe the pyramid shapes in Figure 1c. We can also observe PVDF nanofibers on the surface at an even higher resolution in Figure 1d. The average diameter was about 100–200 nm with a smooth surface. This surface architecture of the single-side or double-side EP-PVDFnfs will significantly improve the output performance, owing to its high dielectric constant of the PVDF and the large contact area of the friction layer. This work successfully prepared elastic, conductive hydrogels from PAAM, PVP, and CaCl_2_. Fourier infrared spectroscopy (FTIR) was used to characterize the molecular structure of PAC hydrogel (Figure 2a). The absorption peaks from the –OH (3300 cm^−1^), –C=O (1653 cm^−1^), and –CH_2_-CH_2−_ (1454 cm^−1^) groups are attributed to their stretching vibrations. The characteristic peak at 1286 cm^−1^ corresponds to the C-N group’s stretching vibration from the PVP molecular segments. The absorption peaks at 1080 cm^−1^ and 1030 cm^−1^ are related to the stretching vibrations of C–C, C–O, or C–O on β-cyclodextrins molecular segment, respectively. The FTIR observations confirm the formation of PAV hydrogels. The mechanical elastic property is shown in Figure 2b. The PAC hydrogel exhibited an excellent elongation of 1241% with a tensile strength at the break of 141.5 kPa, and the single-side EP-PVDFnfs thin film also showed an even higher elongation of 1372% with a tensile strength at the break of 230.5 kPa. Figure 2c shows the electrical stability test of the hydrogel. The resistance variation (*R*/*R*_0_) showed relatively stable during 2000 cycles from 0% to 100% strain at 2 Hz. Figure 2d shows the high-resolution response from the 1000th to the 1010th cycles.

**Figure 3:**
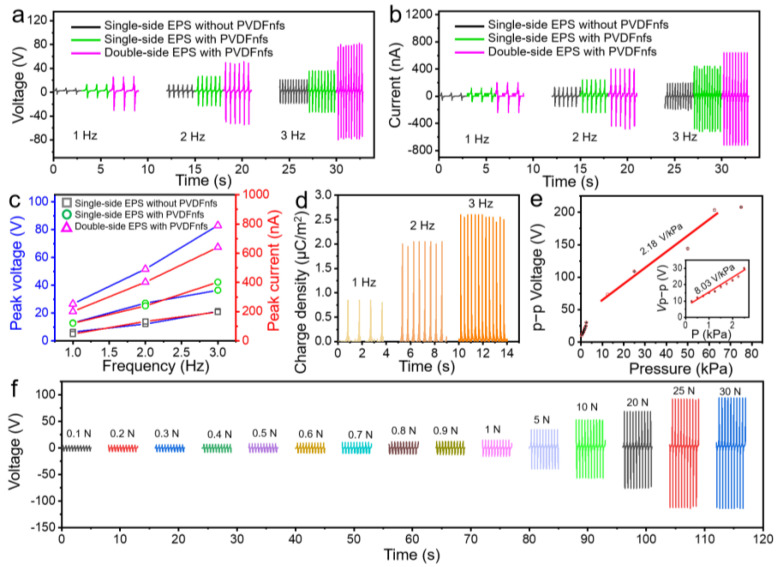
The response voltage of the three as-prepared sensors from the single-side EPS without PVDFnfs, the single-side EPS with PVDFnfs, and the double-side EPS with PVDFnfs is shown in (**a**) output voltage, (**b**) short-circuit current, and (**c**) comparison of the corresponding peak voltage and peak current versus different frequency. The double-side EPS with PVDFnfs sensors of (**d**) charge density at different frequencies, (**e**) the peak to peak response voltage under various pressure from 0.25 kPa to 75 kPa at 2 Hz, and (**f**) their voltage response to the corresponding forces from 0.1 N to 30 N.

To measure the sensors’ electrical output performance, we used commercial rubber as friction-positive material, and we attached the sensors to the pressure head of the universal material test instrument. The effective contact area of the sensors was 2.0 × 2.0 cm^2^, and the air gap was set at 5.0 cm for all the tests. The response voltage and current are shown in Figure 3. All the samples showed a similar trend as the reported references that both the *V*oc and *I*sc of the triboelectric sensors increased with the increasing contact frequency (Figure 3a–c), as shown by Equation (1) [24]:

*I* = *N* × *e* × *s* × *v* (1)

where *I* is the current with external load, *N* is the number of transferred electrons, *e* is the charge of electrons, *s* is the cross-sectional area of electron transport, and *v* is the rate of electron transport. The *v* (electron transfer rate) increases as the tactile frequency increases from 1 Hz to 3 Hz, according to the formula, resulting in the increase in *V*oc (from 2.8 V to 6.3 V) and *I*sc (46.6 nA to 197.4 nA) for the sample of single-side EPS without PVDFnfs. We can see from Figure 3a–c that, for the samples of single-side EPS with PVDFnfs and double-side EPS with PVDFnfs, the peak *V*oc exhibited twice and 4.3 times of the *V*oc and *I*sc than that of the sample of single-side EPS without PVDFnfs, respectively. The results reveal that electrospun PVDF nanofibers have greatly improved the electrical output performance. Subsequently, we studied the charge transfer density versus different frequencies for the samples of double-side EPS with PVDFnfs. From Figure 3d we can observe that the charge transfer density showed 0.85 μC/m^2^, 2.05 μC/m^2^, and 2.60 μC/m^2^ at the increasing of the frequencies from 1 Hz to 3 Hz, respectively. Moreover, the double-side structure also enhanced the output performance more than the samples with single-side structures. The electrical output signal of the double-side EPS with PVDFnfs sensor under various pressure and their corresponding forces under 2 Hz are shown in Figure 3e,f. When the applied forces increased from 0.1 N to 1 N, the observed output *V*_OC_ increased dramatically from 9.8 V to 30.0 V, with a sensitivity of 8.03 V/kPa (20.1 V/N). It may be because of the wrinkled micropyramid arrays’ deformation process. As the applied force increased from 1 N to 30 N, the output performances increased from 38.1 V to 98.6 V, with a sensitivity of 2.18 V/kPa (5.45 N/V), and the output *V*_OC_ became nearly stable. This phenomenon could be because the applied forces of 30 N delivered the maximum contact between the surface and double-side EPS with PVDFnfs sensors.

**Figure 4:**
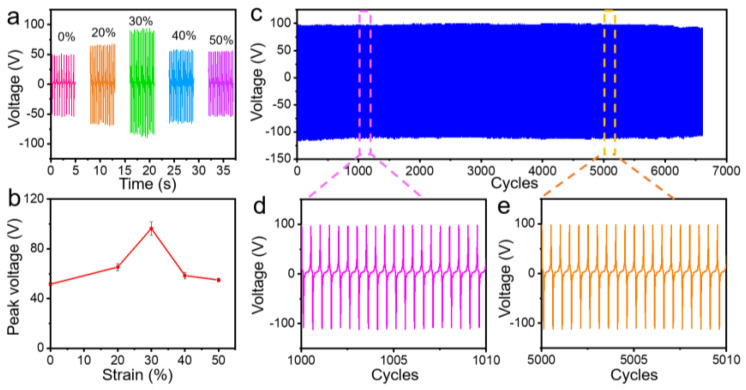
(**a**) The output voltage variation and (**b**) the corresponding peak voltage values of the double-side EPS with PVDFnfs under various stretching strains from 0% to 50% at 2 Hz. (**c**) Stability and durability test of the double-side EPS with PVDFnfs under 13,000 working cycles from 0% to 30% tensile strain at 2 Hz and high-resolution signals of (**d**) the 1000th to the 1010th cycles and (**e**) the 5000th to the 5010th cycles.

We investigated the ductility and the electrical output performance under the different strains of the double-side EPS with PVDFnfs sensor in Figure 4. We observed that the *V*oc increased about twice the 0% strain state when the strain was 30% (96.4 V). During this process, the friction layer became thinner and thinner. Meanwhile, the wrinkled micropyramid arrays deformed more and more as the external stretching of the sensor, resulting in a much closer interface and a larger contact area between the conductive hydrogel and the double-side Ecoflex with PVDFnfs elastic thin film of the sensor, which may greatly benefit the output *V*oc. However, as the stretching strain continued to increase from 30% to 50%, the output *V*oc dropped to 55.0 V, which may occur because of the increasing resistance of the conductive hydrogel. The *V*oc value of the double-side EPS with PVDFnfs was measured during 13,000 cycles and as strain stress from 0% to 20% at a frequency of 2 Hz (Figure 4c–e). The output performance of the triboelectric sensors exhibited excellent durability and stability for the long-time loading–unloading cycles without any noticeable degradation. Table 1 compares the specific performance data such as stretchability, detection limit, and sensitivity, with the recently reported stretchable triboelectric devices. Our device showed quite excellent sensitivities and other performances.

**Figure 5:**
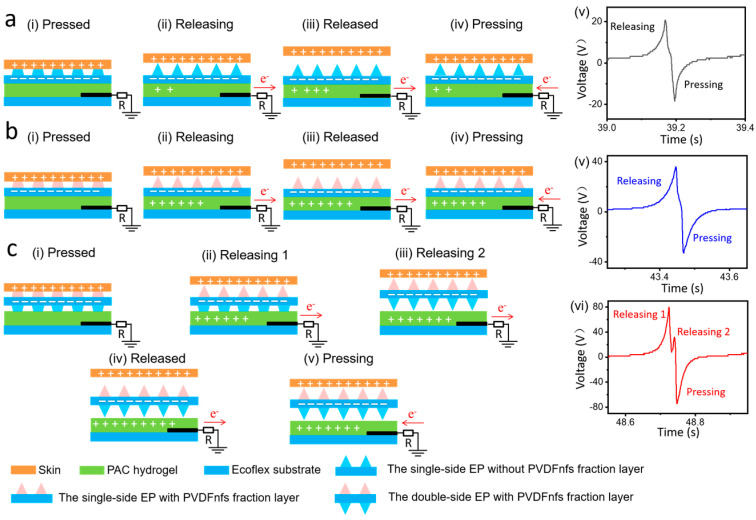
Sketches and the corresponding electrical output voltage curves that illustrate the operating principle of the sensors. (**a**) The single-side EPS with PVDFnfs, (**b**) the single-side EPS with PVDFnfs, and (**c**) the double-side EPS with PVDFnfs.

Based on triboelectrification and electrostatic induction [33], the working principle of the single-side EPS without PVDFnfs, single-side EPS with PVDFnfs, and double-side EPS with PVDFnfs was illustrated in Figure 5a–c, respectively. Take the single-side EPS with PVDFnfs device for example. When our skin touched the sensor, the pressure was applied (Figure 5(a-i)), resulting in the deformation of the wrinkled micropyramid structure of the sensor. Opposite polarity charges coincide on the contacting surface and can be maintained relatively long. By releasing the force, the skin and the sensor were separated, leaving excess positive charges behind on the surface of the Ecoflex thin film. As a result, directed diffusion of the ions occurred in the interior of the sensor as the gradual changing of the electric potential difference (Figure 5(a-ii,a-iii)).

Subsequently, the reapproach of the skin to the sensor induced the reverse of the potential difference, resulting in the opposite direction movement of the electrons (Figure 5(a-iv)). Subsequently, an alternating current was generated to flow through the circuit by repeating the pressing-releasing process. The open-circuit voltage signal of the single-side EPS with PVDFnfs device shown in Figure 5(a-v) proves its working flow. Figure 5b shows the working mechanism of the single-side EPS with PVDFnfs. Owing to the existence of electrospun PVDF nanofibers, the output voltage of single-side EPS with PVDFnfs improved about twice that of the single-side EPS without PVDFnfs (Figure 5(b-i–b-v)). A unique two-peak phenomenon of the electrical output *V*oc was found for the double-side EPS with PVDFnfs in Figure 5(c-v). It revealed a two-step separation during releasing process. The primary peak may be attributed to the deformation recovery of the micropyramid arrays attached to the skin at the moment when the external forces were removed, taken as the process of releasing 1 (Figure 5(c-ii,c-vi)). In contrast, the secondary peak was attributed to the contact separation between the side with micropyramid arrays of the Ecoflex thin film and the conducting hydrogel, taken as the process of releasing 2 (Figure 5(c-iii,c-vi)). This two-step separation process significantly enhanced the electrical output performance.

**Figure 6:**
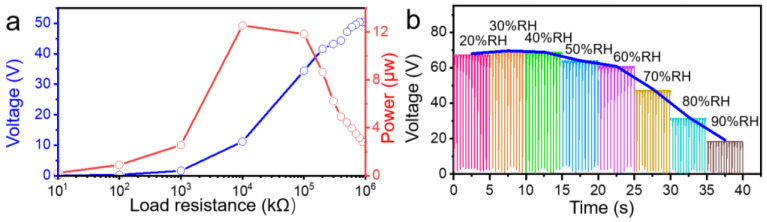
(**a**) Load voltage and peak power curves and (**b**) voltage output under different ambient relative humidity conditions of double-side EPS with PVDFnfs samples.

The peak power and load voltage curve of the double-side EPS with PVDFnfs measured by changing various load resistance at the frequency of 2 Hz, as seen in Figure 6a, show that when the external resistance load was 10 MΩ, the maximum peak power reached 11.8 μW. Different ambient humidity may influence the output performance of the triboelectric sensors because water molecules may adhere to the surface of our devices and form a few nm or sub nm water layer. The water layers are ubiquitous and essential in charge transfer [34]. We tested the output performance of the double-side EPS samples under different relative humidity (RH) conditions from 20%RH to 90%RH in an 80 cm long, 40 cm wide, and 40 cm high closed testing box (shown in Figure 6b). The results showed that the double-sided device displays stable output performance when the relative humidity is 30%RH and 40%RH, and the performance began to decrease when the relative humidity reached 50%RH. Then the output voltage continued to drop as the relative humidity increased to 90%RH. All the other measurements in this work were performed with 60–65%RH at room temperature.

### Human Motion Detection

**Figure 7:**
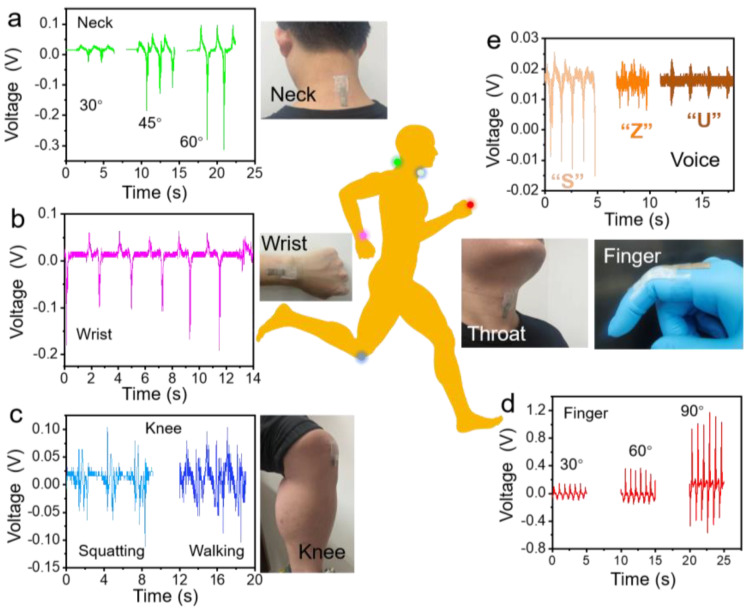
The output voltage and photos of different human motions using the double-side EPS with PVDFnfs sensors attached to the corresponding parts of a volunteer: (**a**) the neck (bent at 30°, 45°, 60°), (**b**) the wrist, (**c**) the knee while walking, (**d**) the index finger (bent at 30°, 60°, 90°), and (**e**) the throat (four times pronouncing “S”, “Z”, and “U”).

We applied the as-prepared double-side EPS with PVDFnfs sensor to different human body parts, and different parts of the joints can exhibit unique output signals. We attached the as-prepared double-side EPS with PVDFnfs sensor to the neck, the knee, the wrist, and the finger, as shown in Figure 7. The excellent flexibility and elasticity can provide better contact and conformability between the sensor and the human skin, significantly affecting its electrical output performance. The differences in the response *V*oc included the shapes, response/recovery time, and the peak value among various joint movements. Our sensor can accurately monitor the neck (Figure 7a), wrist (Figure 7b), and knee (Figure 7c) for pretty large joint motions, such as nodding the head up and down, bending the wrist, and switching walking and squatting states. Significant *V*oc signals can be observed even from a tiny joint bending, such as index finger movements from angles of 30° to 90° (in Figure 7d). Moreover, we attached the sensor to a volunteer’s throat to demonstrate the reorganization of the vocal cord’s vibration. The obtained dynamic output *V*oc can be seen when the volunteer pronounced “S”, “Z”, and “U” in Figure 5e. The as-prepared triboelectric sensors showed the ability to monitor multiple human physiological activities, which is essential for real-time fast posture/action recognition and medical rehabilitation.

## 4. Conclusions

In summary, we reported a stretchable, electrospun nanofibers composed wrinkled micropyramidal architectures-based self-powered triboelectric nanogenerator, which was composed of the PAAm/PVP/CaCl_2_ conductive hydrogel as the electrode and elastic Ecoflex thin films with wrinkled micropyramidal architectures as the friction layer. The different surface modification strategies of the friction layer have been studied, including electrospun PVDF nanofibers surface modification and single-side and double-side wrinkled micropyramidal architectures. The double-side EPS with PVDFnfs sensors exhibited excellent tactile and strain sensing performance, long-term durability, and were demonstrated as epidermal sensors to monitor various human body’s movements. Those features give the as-prepared triboelectric sensors high potential in wearable electronics and intelligent robots.

## Figures and Tables

**Table 1:** Comparison of the reported stretchable triboelectric devices.

Device Composition	Stretchability	Detection Limit	Sensitivity	Ref.
PDMS/HTS-c-hydrogel	900%	1 kPa	n/a	[25]
PDMS/PAAm hydrogel	330%	n/a	n/a	[26]
VHB/PAAm hydrogel	1160%	1.3 kPa	n/a	[27]
PDMS/PAMPS ionogel	<300%	0.1N	1.76 V/N	[28]
SA−Zn hydrogel/Ecoflex	830%	n/a	6.989 V/N	[29]
PTFE/BTO nanocube/PAM hydrogel	800%	0.25N	7.91 V/N	[30]
PDMS/EGaIn	50%	0.23 Pa	0.239 V/kPa	[31]
PDMS-PU0.6-PA0.4-Zn/PDMS-PU0.6-PA0.4-Zn-NSP	10000%	0.25 kPa	4.209 V/kPa	[32]
Ecoflex/PVDF nanofibers/PACC hydrogel	1370%	0.25 kPa (0.1 N)	20.1 V/N (8.03 V/kPa)	This work

## Data Availability

The data presented in this study are available on request from the corresponding author.
